# Variably severe systemic allergic reactions after consuming foods with unlabelled lupin flour: a case series

**DOI:** 10.1186/1752-1947-8-55

**Published:** 2014-02-16

**Authors:** Amolak S Bansal, Mihir M Sanghvi, Rhea A Bansal, Grant R Hayman

**Affiliations:** 1 Department of Immunology and Allergy, St Helier Hospital, Carshalton, Surrey SM5 1AA, UK

**Keywords:** Anaphylaxis, Food labelling, Gluten-free spaghetti, Lupin allergy, Oral allergy

## Abstract

**Introduction:**

Lupin allergy remains a significant cause of food-induced allergic reactivity and anaphylaxis. Previous work suggests a strong association with legume allergy and peanut allergy in particular. Both doctors and the public have little awareness of lupin as an allergen.

**Case presentation:**

Case 1 was a 41-year-old Caucasian woman without previous atopy who developed facial swelling, widespread urticaria with asthma and hypotension within minutes of eating a quiche. Her lupin allergy was confirmed by both blood and skin tests. Her lupin sensitivity was so severe that even the miniscule amount of lupin allergen in the skin testing reagent produced a mild reaction.

Case 2 was a 42-year-old mildly atopic Caucasian woman with three episodes of worsening urticaria and asthma symptoms over 6 years occurring after the consumption of foods containing lupin flour. Blood and skin tests were positive for lupin allergy.

Case 3 was a 38-year-old Caucasian woman with known oral allergy syndrome who had two reactions associated with urticaria and vomiting after consuming foods containing lupin flour. Skin testing confirmed significant responses to a lupin flour extract and to one of the foods inducing her reaction.

Case 4 was a 54-year-old mildly atopic Caucasian woman with a 7 year history of three to four episodes each year of unpredictable oral tingling followed by urticaria after consuming a variety of foods. The most recent episode had been associated with vomiting. She had developed oral tingling with lentil and chickpeas over the previous year. Skin and blood tests confirmed lupin allergy with associated sensitivity to several legumes.

**Conclusions:**

Lupin allergy can occur for the first time in adults without previous atopy or legume sensitivity. Although asymptomatic sensitisation is frequent, clinical reactivity can vary in severity from severe anaphylaxis to urticaria and vomiting. Lupin allergy may be confirmed by skin and specific immunoglobulin E estimation. Even skin testing can cause symptoms in some highly sensitive individuals. The diagnosis of lupin allergy in adults may be difficult because it is frequently included as an undeclared ingredient. Better food labelling and medical awareness of lupin as a cause of serious allergic reactions is suggested.

## Introduction

Lupin flour allergy can be severe and unpredictable and despite European legislation (Commission Directive 2006/142/EC) introduced in December 2006 it is often still used without disclosure on the list of ingredients in foods such as pastries. There is significant cross-reactivity between lupin and peanut but less so with other legumes [1]. We describe four cases of severe lupin flour allergy in adults with and without associated legume sensitisation. The absence of adequate food labelling and poor public and physician awareness of lupin as a cause of severe allergic reactivity led to a marked delay in the diagnosis and required detailed investigation to uncover the cause of the reactions.

## Case presentations

### Case 1

A 41-year-old Caucasian woman ate approximately 150g of a superior range quiche. Within a minute she felt a tickling sensation in her throat and eyes and the latter became swollen. She became dyspnoeic with tightness of her chest, wheezing and coughing. Examination in hospital confirmed a tachycardia of 120/minute, blood pressure of 75/50mmHg and scattered wheezes throughout both lungs. She then rapidly developed widespread urticaria and further swelling of her face. Treatment with adrenaline, antihistamines and steroids produced gradual improvement over the ensuing 4 hours and she required two further injections of adrenaline.

Subsequent questioning confirmed the absence of previous atopy, concurrent use of antacids/proton pump inhibitors or non-steroidal anti-inflammatory drugs, suggestion of the oral allergy syndrome (OAS) or features to suggest a food exercise anaphylaxis. All foods eaten on the day of the reaction had been eaten before on numerous occasions without reactivity.

Skin testing was performed to include foods that may comprise undisclosed ingredients. This confirmed a very large wheal to lupin flour at 11mm but with negative responses to milk, egg, wheat, soya, sesame seed, peanuts, cod, prawn, salmon, beans, beef, pork as well as to mixed grass, tree and weed pollens. However, soon after the skin testing the patient developed tickling of her throat that responded only slowly to oral antihistamines. On return home she vomited and then improved gradually.

Her blood tests using the ImmunoCAP™ assay (Thermo Scientific, Uppsala, Sweden) later showed an isolated sensitivity to lupin flour with a specific immunoglobulin E (IgE) of 25.4kUA/L and with a total IgE of 204kU/L. There were negative responses to peanut, sesame seed and salmon as well as to 114 allergens tested in the ImmunoCAP ISAC® (Immuno Solid-phase Allergen Chip) (Thermo Scientific, Uppsala, Sweden) panel including Ara h 1. Further enquiry confirmed that the quiche contained lupin flour in the pastry crust. This was not mentioned in the list of ingredients.

### Case 2

A 42-year-old Caucasian woman had three reactions over 6 years. Each was delayed by 10 minutes or more after consumption of the suspected lupin-containing food and associated with generalised urticaria and asthma but without hypotension, vomiting and diarrhoea. The first occurred with a small piece of cake in an upmarket bakery, the second occurred 3 years previously and with a small piece of bread from a health food shop and the third reaction occurred with a small amount of short crust pastry. With the latter reaction the asthma symptoms were sufficiently severe to require emergency treatment in hospital with nebulised salbutamol, intramuscular antihistamines and hydrocortisone. All the foods were later discovered to contain lupin flour as a minor ingredient. The patient denied symptoms of an underlying OAS, nut allergy, legume sensitivity and seasonal allergic rhinitis. She had mild asthma induced by exposure to animal dander and mild eczema evident with stress.

Skin prick testing confirmed an 8mm wheal to a commercial lupin flour reagent, a 4mm wheal to peanut and negative responses to soya, white bean, sesame seed and wheat. Her blood tests using the ImmunoCAP™ assay showed a total IgE of 467kU/L and she had 75kUA/L specific IgE to lupin flour with 4.04kUA/L to peanut, 9.6kUA/L to Ara h 2, 6.2kUA/L to Ara h 8, 5.4kUA/L to Bet V1, 1.61kUA/L to fenugreek and <0.1kUA/L to soya, Ara h 1 and Ara h 3.

### Case 3

A 38-year-old Caucasian woman experienced immediate oral and throat itching followed, 1 hour later, by generalised urticaria and vomiting after eating pancakes and cooked cherries. There were no respiratory or cardiovascular symptoms. She had a history of atopic disease since childhood with atopic eczema, seasonal allergic rhinitis and mild asthma. She also had features of the birch pollen-related OAS, experiencing oral itching when eating raw apples, melons, hazelnut and plums.

Initial skin prick tests (SPTs) were positive to mixed grass pollens, mixed tree pollens, silver birch pollen, walnut, hazelnut and potato. However, she then had an episode of generalised urticaria 1 hour after eating gluten-free spaghetti containing lupin flour. Further SPTs were negative to raw and cooked cherries but were significantly positive with 5mm and 7mm wheals to the spaghetti containing lupin and to lupin flour extract respectively. Further investigation confirmed the pancakes to contain lupin flour also. Unfortunately she has been unable to attend for follow-up and thus further assessment of an underlying legume sensitisation has not been possible. However, she did not have any indication of legume sensitivity in her clinical history.

### Case 4

A 54-year-old Caucasian woman with mild seasonal allergic rhinitis had for the previous 7 years had three to four episodes each year of unpredictable oral tingling followed by urticaria after consuming a variety of foods. The latter generally included high-end pastries and bread but without exercise, alcohol or drug ingestion. Her most recent episode had been associated with vomiting. None of the reactions had been associated with cardiorespiratory compromise or diarrhoea. Over the previous few months she had developed mild oral tingling with lentil and chickpeas but these symptoms were brief and she had continued to consume these. Skin testing confirmed a 7mm wheal to lupin flour but with negative responses to milk, wheat, peanut, white beans, soya and sesame seeds. Blood tests confirmed a mildly raised total IgE of 201kU/L with a highly positive specific IgE to lupin at 55kUA/L. The specific IgE to fenugreek was positive at 5.3kUA/L, chickpeas at 4.2kUA/L and lentil at 3.1kUA/L. The results were negative to pea, soya, whole peanut and Ara h 1. Since careful avoidance of lupin-containing foods she had not had any reactions for the previous 2 years.

## Discussion

Lupin flour is derived from *Lupinus angustifolius* and is a legume. Its use, particularly in baked products, has increased significantly over the last decade. This may relate to its high protein content and its possible ability to lower cholesterol levels and improve glycaemic control [2]. The major allergenic proteins of lupin are not fully characterised but include α- and β-conglutins, with a lesser presence of γ- and δ-conglutins [3]. The β-conglutin corresponding to Lup an 1, which is considered the major lupin allergen [2], has sequence similarities to the peanut Ara h 1 [3]. Of interest, however, specific IgE to Ara h 1 was negative in patients one and two. The mechanism of lupin sensitisation in our patients without any legume or peanut sensitivity is unclear. However, the recent introduction of lupin flour in foods may represent a novel protein and thus reactivity may represent a new allergy for the patient.

Sensitisation to lupin flour is significantly more frequent than clinical reactivity [4,5]. In both children and adults, lupin sensitisation is more frequent in those with peanut allergy (17.1% and 14.6% respectively) and those with underlying atopy (2.5% and 3.7% respectively) [5]. Others have reported significantly higher rates of sensitisation in those with peanut allergy [6,7]. In our practice we have found lupin sensitisation in approximately one-fifth of our patients with peanut allergy and with only a few having mild and localised clinical reactivity. Clearly this creates problems on how to advise patients. We suggest that lupin-sensitised patients without evidence of any sort of previous reactivity continue on a normal diet but if eating out or consuming unfamiliar foods they should have antihistamines available. With the development of even mild oral itching with lupin contaminated foods, then this should be avoided completely. These patients may benefit from a double-blind oral lupin challenge if there is doubt about clinical reactivity.

In regard to our four cases of lupin allergy, our second patient had a mildly positive SPT to peanut and with positive specific to peanut and to the Ara h 2 and Ara h 8 proteins but not to the Ara h 1 or Ara h 3. This appears to suggest a coincidental nut allergy and a possible underlying OAS. The negative results to Ara h 1 makes cross-reactivity with the lupin Lup an 1 protein very unlikely. In addition, the absence of specific IgE to Ara h 3 makes the peanut sensitivity unlikely to be due to a legume-based cross-reactivity. However, our fourth patient had positive specific IgE to fenugreek, chickpeas and lentil and evidence of a mild clinical legume allergy.

The amount of lupin flour that can elicit a discernable reaction varies considerably and may be as low as 0.5mg [7]. In adults working with lupin flour, sensitisation and clinical reactivity has been reported to cause rhinitis, asthma and conjunctival irritation [8]. Lupin allergy in adults can be very severe and often without previous atopy [9]. Indeed in our first patient without concurrent atopy even the skin testing produced symptoms in the throat and later vomiting. The frequency of lupin allergy in adults with an underlying OAS is not known but was evident clinically in the third patient and suggested serologically on blood tests with the second patient.

In comparison to the European Continent, lupin is a rarely used ingredient in the United Kingdom. When used it is often a minority (<10%) ingredient often in conjunction with wheat flour. The reactions in all our patients occurred after the consumption of wheat products that were later discovered to contain lupin flour. Knowledge about lupin allergy is poor even though 8% of those with peanut allergy may have a potential lupin allergy.

Our first patient demonstrated lupin flour-induced anaphylaxis without prior atopy. This was first reported in children by Wassenberg and Hofer in 2007 [10]. Among the 1522 patients with suspected food allergy reported by Hieta *et al*. [9], 25 (1.6%) were SPT positive for lupin and seven of these patients manifested varying degrees of clinical reactivity. Our second case had two significant allergic reactions of unknown cause before lupin allergy was diagnosed. In the fourth case, three to four reactions had occurred annually for 7 years before lupin allergy was diagnosed. It is therefore important that testing for lupin sensitivity is undertaken in all patients presenting with severe allergic reactions without obvious cause. As with the case reported by Rossi *et al*. [11], the reaction in our third case involved lupin flour used in a gluten-free product. It is vitally important therefore that: a) clinicians are aware of lupin flour as a potential allergen, b) the general public are further educated as to the usage of lupin flour particularly in baked goods and the particular risk posed to those who have a peanut and legume allergy, and c) that manufacturers ensure that any amount of lupin flour (even if masked by wheat flour) is included on lists of ingredients such that affected individuals can take necessary steps to minimise their risk.

## Conclusions

Lupin allergy without previous atopy or legume sensitivity can present for the first time in adult life and vary in severity from asymptomatic sensitisation to severe anaphylaxis. The more expensive high-end wheat-based foods are most frequently incriminated in allergic reactions. Skin prick testing and analysis of specific IgE can readily confirm lupin allergy and a high index of suspicion should be maintained because lupin is frequently used without being listed in the ingredients. In highly sensitised individuals, even skin testing can cause symptoms. Legislation to enforce the declaration of lupin as an ingredient and improved medical awareness of lupin as a cause of serious allergic reactions is suggested.

## Consent

Written informed consent was obtained from the patients for publication of this manuscript and accompanying images. A copy of the written consents is available for review by the Editor-in-Chief of this journal.

## Abbreviations

IgE: Immunoglobulin E; OAS: Oral allergy syndrome; SPT: Skin prick test.

## Competing interests

Each of the four authors declares no conflict of interest in the submission of this report.

## Authors’ contributions

ASB and GRH saw the patients, managed them clinically and drafted the report. RAB and MMS did the literature search and helped write the report. All authors have read and approved the final manuscript.

## References

[B1] Moneret-Vautrin DA Guérin L Kanny G Flabbee J Frémont S Morisset M Cross-allergenicity of peanut and lupine: the risk of lupine allergy in patients allergic to peanuts J Allergy Clin Immunol 1999 104 4 Pt 1 883 888 10518837 10.1016/s0091-6749(99)70303-9

[B2] Guillamón E Rodríguez J Burbano C Muzquiz M Pedrosa MM Cabanillas B Crespo JF Sancho AI Mills EN Cuadrado C Characterization of lupin major allergens (*Lupinus albus L.*) Mol Nutr Food Res 2010 54 11 1668 1676 10.1002/mnfr.200900452 20461737

[B3] Sanz ML de Las Marinas MD Fernández J Gamboa PM Lupin allergy: a hidden killer in the home Clin Exp Allergy 2010 40 10 1461 1466 10.1111/j.1365-2222.2010.03590.x 20701610

[B4] de Jong NW van Maaren MS Vlieg-Boersta BJ Dubois AE de Groot H Gerth van Wijk R Sensitization to lupine flour: is it clinically relevant? Clin Exp Allergy 2010 40 10 1571 1577 10.1111/j.1365-2222.2010.03496.x 20412139

[B5] Gayraud J Mairesse M Fontaine JF Thillay A Leduc V Rancé F Parisot L Moneret-Vautrin DA The prevalence of sensitization to lupin flour in France and Belgium: a prospective study in 5,366 patients, by the Allergy Vigilance Network Eur Ann Allergy Clin Immunol 2009 41 1 17 22 19496348

[B6] Peeters KA Koppelman SJ Penninks AH Lebens A Bruijnzeel-Koomen CA Hefle SL Taylor SL van Hoffen E Knulst AC Clinical relevance of sensitization to lupine in peanut-sensitized adults Allergy 2009 64 4 549 555 10.1111/j.1398-9995.2008.01818.x 19076544

[B7] Shaw J Roberts G Grimshaw K White S Hourihane J Lupin allergy in peanut-allergic children and teenagers Allergy 2008 63 3 370 373 18028245 10.1111/j.1398-9995.2007.01568.x

[B8] Campbell CP Jackson AS Johnson AR Thomas PS Yates DH Occupational sensitization to lupin in the workplace: occupational asthma, rhinitis, and work-aggravated asthma J Allergy Clin Immunol 2007 119 1133 1139 10.1016/j.jaci.2007.01.032 17379286

[B9] Hieta N Hasan T Mäkinen-Kiljunen S Lammintausta K Lupin allergy and lupin sensitization among patients with suspected food allergy Ann Allergy Asthma Immunol 2009 103 3 233 237 10.1016/S1081-1206(10)60187-1 19788021

[B10] Wassenberg J Hofer M Lupine-induced anaphylaxis in a child without known food allergy Ann Allergy Asthma Immunol 2007 98 6 589 590 10.1016/S1081-1206(10)60741-7 17601275

[B11] Rossi G Amato S Mistrello G Gluten-free food as source of hidden allergen (lupine) Eur Ann Allergy Clin Immunol 2009 41 4 123 125 19877566

